# Discourse on the Life, Character, and Services, of Dr. Daniel Drake

**Published:** 1853-03

**Authors:** S. D. Gross

**Affiliations:** Professor of Surgery


### WESTERN JOURNAL

OF

### MEDICINE AND SURGERY.

MARCH, 1853.

ART. I.—*Discourse on the Life, Character, and Services of Dr. Daniel Drake.*—Delivered, by request, before the Faculty and Medical Students of the University of Louisville, Ja nuay 27, 1853 By S. D. GROSS, M. D., Professor of Surgery.

[CONCLUDED.]

Genius has its infirmities, as well as its weaknesses. The world, looking merely at the surface of a man's character, without comprehending the machinery of his mind, and the motives and circumstances which impel it to action, is often incapable of appreciating his feelings, his sentiments, and his wants. The sun, although constantly emitting the same amount of light, is often obscured by clouds, which obstruct his rays, and impair his genial influence. Man, true and faithful to his nature, cannot always smile; his temper is often ruffled, and his mind is often overcast by sadness. He cannot be the same at all times, or to all men. Human nature is human nature in all classes and conditions; now serene and composed, now agitated by storms and passions, approximating man, in the one case, to an angel, in the other to a fiend. In studying the character of men we should never lose sight of the fact that they are but men; nor forget the circumstances in which they are placed, and move, and have their being, nor the ends and designs which they aim and strive to accomplish. Dr. Drake had his foibles and even his faults; but it may be truly asserted that few men, engaged in the turmoil of a public life, were more free from the former or more exempt from the latter. Now that he is gone, let us forget both, and only remember his resplendent virtues.

Dr. Drake never had a vice. His enemies cannot point to a single act of his life, in which there was the slightest approximation to any exhibition of the kind. His moral character was cast in the finest and purest mould. He could not have been bad. His conscientiousness and love of approbation were too large to admit of it. The attachment and reverence which he cherished for his parents were opposed to every feeling of licentiousness and immorality. Their early training produced an impression upon his mind which neither time nor circumstances could efface, but which steadily grew with his growth and strengthened with his strength. His conduct, in all the periods and phases of his life, was squared by the strictest rules of honesty, and by the nicest regard for the feelings and rights of others. Although he was long poor, he always paid his debts to the uttermost farthing. "Pay what thou owest" was with him a golden maxim.

For public amusements he had not only no love, but they were eminently repulsive to his tastes and feelings. The impression made upon his tender mind at May's Lick, by this species of life on parade and gala days, among his father's neighbors, was indelible. He never played a game of cards in his life; gambling and gamblers he alike detested. His whole career, in fact, from its commencement to its close, was an exhibition of attachment to moral principle. His life was one of constant and untiring industry and exertion, exhausting meditation, and the most resolute self-denial. He never knew any thing of the luxury of the chase, or of trout-fishing. He might have read Isaak Walton's "Complete Angler," the "Contemplative Man's Recreation," or "Salmonia," or "Days of Fly-fishing," with a view of seeking refuge from ennui, or relaxation from his scientific labors; but never for the purpose of learning, much less practising, the art. In a word, he labored incessantly under the impulse of a lofty ambition, and under an intense desire to improve his profession and benefit mankind.

We have said nothing, as yet, of Dr. Drake, as he appeared in his domestic relations, in the bosom of his own family, among his children and grand-children, and at his own hearth. It is no idle curiosity which prompts us to lift the veil of private life, and to look, for a moment, into the very caverns and recesses of the heart of our departed friend. We have already seen how fond he was of her who, for nearly twenty years, had shared his joys and his sorrows; how tenderly he loved her, and how faithfully he cherished her memory after her death. His attachment to her never faltered; it was deep, firm, and abiding, inrooted in the very fibres of his heart. It was sublime!

To say that he was a tender, devoted, and exemplary father would but ill express the truth; he was more; his love ran in the deepest and broadest channel. He idolized his children and watched over their interests and happiness with the care and solicitude with which the eagle watches over his young. His affection for them was omnipresent, and all-absorbing. The numerous letters which he addressed to them during his long winter residences in this city, and during his travels in various sections of the Union in search of materials for his great work, to say nothing of his frequent visits to Cincinnati, amply attest the truth of this remark. It seemed as if the caldron of his affection were constantly boiling over, and seeking vent in such missives. They were to him as winged messengers, bearing glad-tidings from his own heart to the hearts of his children and grand-children. His correspondence with the different members of his family would cover many volumes ; for it was not only frequent, but copious, extending often through many pages. His grand-children came in for a full share of this kind of intercourse, so honorable to the heart and head of this great and good man. Their birth-days never failed to be hailed by a letter, generally abounding in some witticism, some simple anecdote, or some good advice, conveyed in a plain, agreeable, and tender style, well adapted to their comprehension. During his sojourn in our city, he paid them every winter not less than three or four visits, often lecturing twice, and, sometimes, even thrice a day, that he might get in advance of his course, and thus obtain the requisite time. He had not a colleague of whom he did not occasionally borrow an hour for this purpose.

In connection with this branch of the subject may be mentioned a series of letters which Dr. Drake addressed to his children, a few years ago, while engaged in delivering a course of medical lectures in this city. They extend over several hundred pages of manuscript, and have lately, since his death, been bound in a quarto volume, under the title of "Reminiscential Letters." I am sure that every one who peruses them will, like myself, regard them as a most precious legacy from a doting father to his devoted childred and grand-children. They recount, in glowing terms, and with a true Daguerreotype-likness, the deeds and scenes of his childhood and boyhood up to the time he entered Cincinnati as a student of that profession which he was destined so much to adorn and illustrate by his labors, his teachings, and his writings.

There is not an occupation incident to a new settlement in the West, or in which he was himself engaged, which he does not pourtray in these epistles in the most vivid and graphic manner. Had he been able to wield the pencil of a Cole, he could not have painted, in truer colors, the voyage of the first fifteen years of his monotonous but not uneventful life. A selection, by some judicious relative, or friend of the deceased, would form a valuable contribution to our literature, especially for the young, whose minds could not fail to be benefitted and improved by its perusal. In point of charm and interest, in moral and religious tone, in filial reverence and devotion, in just and philosophical deduction, they are not surpassed by the beautiful Autobiography of Jean Paul, as it is exhibited in the life of that extraordinary man by Mrs. Lee, herself one of the most delightful of writers.

The life of Dr. Drake was eminently eventful. No man that our profession has yet produced has led so diversified a career. He was, probably, connected with more medical schools than any individual that ever lived. It is rare that physicians interest themselves in so many public and professional enterprises as he did. His mind was of unlimited application. His own profession, which he served so well and so faithfully, was incapable of restraining it; every now and then it overleaped its boundaries, and wanderd off into other spheres. His career, in this respect, affords a remarkable contrast with that of medical men generally, whose pursuits furnish few incidents of public interest or importance. His mission to his profession and to his age was a bright and happy one. No American physician has performed his part better, or left a richer savor along his life-track.

But his life was not only eventful; it was also eminently laborious. No medical man ever worked harder, or more diligently and faithfully; his industry was untiring, his perseverance unceasing. It was to this element of his character, blended with the intensity we have described, that he was indebted for the success which so preëminently distinguished him from his professional co-temporaries. He had genius, it is true, and genius of a high order, but without industry and perseverance it would have availed him little in the accomplishment of the great aims and objects of his life. He seemed to be early impressed with the truth of the remark of Seneca: "Non est ad astra mollis á terris via." He felt that he did not belong to that fortunate class of beings whose peculiar privilege it is to perform great enterprises without labor, and to achieve great ends without means. His habits of industry, formed in early boyhood, before, perhaps, he ever dreamed of the destiny that was awaiting him, forsook him only with his existence. His life, in this respect, affords an example which addresses itself to the student of every profession and pursuit in life, which the young man should imitate, and the old man not forget.

There was one trait in his character of which I have not yet spoken, and which I approach with much diffidence. I allude to his humility. So largely did this enter into his conduct and character, that I cannot, for a moment, suppose that it was not real and genuine. From what I saw of it, in the different circumstances of his life, it appeared to me as if it had been deeply inlaid in his very constitution, and that it was, therefore, compelled, not unfrequently, to exhibit itself in his conduct and conversation. What corroborates this opinion is that in his "Reminiscential Letters," already more than once alluded to, he speaks of the low state of his pride. "That passion," he remarks, "was, indeed, never strong; and, moreover, was counterpoised by a humility, which always suggested how far short I came of the excellence which ought to be attained. With these traits," he continues, "if I had been born a slave, I should never have become a rebel; but conforming to my condition, and rendering diligent service, have acquired the confidence of my master."

Dr. Drake never visited Europe. In the many conversations which passed between us on this subject, he invariably assigned, as a reason for not going, his literary and scientific deficiencies, as if he feared to come in contact with his transatlantic brethren, whom he supposed to be so much more enlightened than himself. I never conld doubt the sincerity of his declaration. That he would have been a worthy representative of our profession abroad, none that knew him can doubt.

He always had an humble estimate of his scientific labors. When the first volume of his great work was published, he was naturally very anxious as to the manner in which it would be received by the profession. Almost the first notice of it that appeared was read in his presence at the meeting of the American Medical Association at Cincinnati, in 1850, by Dr. Stillè, chairman of the Committee on Medical Literature, and had the effect of completely overpowering him. He felt that the work was safe, and humility baptized his triumph in a flood of tears.

The great defect in his character was *restlessness*, growing, apparently, out of his ardent and impulsive temperament, which never permitted him to pursue any subject very long without becoming tired of it, or panting for a change. His mind required diversity of occupation, just as the stomach, to be healthful, requires diversity of food. Hence, while engaged in the composition of his great work, he could not resist the frequent temptations that presented themselves to divert him from his labors. His delight was to appear before the public, to deliver a temperance address, to preside at a public meeting, or to make a speech on the subject of internal improvement, or the Bible or missionary cause. For a similar reason he stepped out of his way to write his letters on Slavery, and his

Discourses before the Cincinnati Medical Library Association. No man in our land could have done these things better, few, indeed, so well; but, useful as they are, it is to be regretted that he undertook them, because they occupied much of his time that might, and, in the opinion of his friends, ought to, have been devoted to the composition and completion of his great work, the ultimate aim and object of his ambition. Like Adam Clarke, he seemed to think that a man could not have too many irons in the fire, and the consequence was that he generally had the tongs, shovel, and poker all in at the same time.

It was the same restless feeling that caused his frequent resignations in medical institutions. Had his disposition been more calm and patient, he would have been satisfied to identify himself with one medical school, and to labor zealously for its permanency and renown. In moving about so frequently, he induced people to believe that he was a quarrelsome man, who could not agree with his colleagues, and whose ruling passion was to be a kind of autocrat in every medical faculty with which he was connected. But, while his own conduct gave coloring to such an idea, nothing could have been more untrue.

But Dr. Drake was not merely a physician, a teacher, and an author. He was more; he was a Christian, and a Christian from choice and conviction, not from motives of expediency and self-interest. A reverence for the Deity had been deeply implanted in his very constitution; and the pious teachings of his parents, in his early childhood, were never effaced from his mind.

In 1840, he united himself in this city with St. Paul's church, then under the pastoral charge of that excellent and holy man, the late Rev. William Jackson. He had long contemplated such a step, and as the time approached for its consummation he looked forward to the event with feelings of no ordinary satisfaction. His wish was to be brought into closer fellowship with his christian brethren, and to seal his affection for the church with the blood of a dying Savior. He was ever afterwards a faithful, consistent, and untiring attendant upon public worship. Nor was he a less regular observer of the daily duties of private and family devotion. The charge of irreligion, sometimes urged against medical men, never received any confirmation from his character.

His mind was deeply impressed with the evangelical truths of the gospel, and he could not contemplate, without serious apprehensions, the High Church movements and tendencies of the present day. He saw that Puseyism had taken deep root in the minds of many good people, and he considered it a sacred duty to aid, if possible, in counteracting what he regarded as its evil influences. So much did he have this subject at heart that he was induced, only a few years before his death, to discuss it, at some length, in the Philadelphia Episcopal Recorder, in a series of articles written with much judgment and ability. They appeared under the signature of a "Western Layman," and attracted much attention at the time. At the period of his death, he was under an engagement to furnish a series of articles for a new Review, about to be established by the leaders of the "Low Church" party.

Nearly all the early settlers of May's Lick were baptists, and hence all his early ideas of Christian doctrine, worship, and deportment were derived from that denomination. He would probably have attached himself to that church, had he not been deterred by the fact that it has no written creed, and no system of government beyond the democracy of the congregation. When only six years of age, and when he was yet hardly able to read, a baptist catechism was put into his hands by the Rev. Mr. Wood, an old clergyman, of Washington, Kentucky. It opened with the doctrines of the Trinity, which so perplexed him that he ever afterwards retained a prejudice against all similar publications. The first Episcopal Church of Cincinnati was organized at his house. *

He was well read in the Bible; and his acquaintance with theological literature was by no means inconsiderable. He was familiar with the tenets of the principal denominations which compose the Christian world; and, while he was tolerant of other sects, he had a warm and decided leaning towards his own.

Every thing relating to a distinguished man acquires an interest, which produces a desire to become acquainted with his personal history and appearance; even to the most trifling circumstance. We wish to know how he looked, and walked, and talked; how he deported himself in the social circle, in the drawing-room, and in the presence of his friends and neighbors; how he amused himself, and spent his time; how he studied; and what means he employed to make himself just the man he was. The little sayings and doings of a great man are always objects of interest, and frequently throw more light upon his real character than his public acts. Our interest in Socrates is heightened ten fold by our knowledge of Xantippe. We can almost forgive the sentence which condemned him to swallow the fatal draught: but we can hardly excuse the conduct of a wife who destroyed the domestic happiness of her husband.

In regard to our friend, his personal appearance was striking and commanding. No one could approach him, or be in his presence, without feeling that he was in contact with a man of superior intellect and acquirement. His features, remarkably regular, were indicative of manly beauty, and were lighted up and improved by blue eyes of wonderful power and penetration. When excited by anger, or emotion of any kind, they literally twinkled in their sockets, and he looked as if he could pierce the very soul of his opponent. His countenance was sometimes staid

* Reminiscential Letters.

and solemn; but generally, especially when he was in the presence of his friends, it was radiant and beaming. His forehead, though not expansive, was high, well-fashioned, and eminently denotive of intellect. The mouth was of moderate size, the lips of medium thickness, and the chin rounded off and well-proportioned. The nose was prominent, but not too large. The frosts of sixty-seven winters had slightly silvered his temples, but had made no other inroad upon his hair. He was nearly six feet high, rather slender, and well-formed.

His power of indurance, both mental and physical, was extraordinary. He seemed literally incapable of fatigue. His step was rapid and elastic, and he often took long walks, sufficient to tire men much younger, and, apparently, much stronger than himself. He was an early riser, and was not unfrequently seen walking before breakfast with his hat under his arm, as if inviting the morning breeze to fan his temple and cool his burning brain.

No examination was made of his brain after death. Had this organ been placed in the scale, its weight would have fallen far below that of the brain of Cuvier, and of Webster; but if it could have been analysed by the chemist and the microscopist, its finer texture would have been found fully equal to that of either of these illustrious individuals. It is the mind that makes the man, and the structure, not the weight of the brain, that makes the mind. A person may have an enormous brain, and yet be an idiot; and, on the other hand, this organ may be unusually small, and yet the intellect over which it presides be astonishingly great, and of the noblest quality. The engine of Dr. Drake's brain was, to borrow a not inappropriate expression, of the finest manufacture, and our regret is that it cannot be put into some new hull for further service.

His manners were simple and dignified; he was easy of access, and eminently social in his habits and feelings. His dress, and style of living were plain and unostentatious. During his residence in Cincinnati, previously to his connection with this University, his house was the abode of a warm but simple hospitality. For many years, no citizen of that place entertained so many strangers and persons of distinction.

In politics he was a Whig, and never failed to exercise his elective franchise. During the presidential canvass of 1840, in which his early friend, the late General Harrison, himself at one time a student of medicine, was the Whig candidate, Dr. Drake evinced a deeper interest, and took a more active part, than he ever did before, or afterwards, in any contest of a similar kind. He was the ardent friend of rational liberty throughout the world; and no man ever gloried more in the institutions of his country.

He was naturally conscientious. A desire to execute every trust that was confided to him, promptly and faithfully, formed a prominent trait in his character. He was always unhapyy, if, through neglect, inadvertence, or misfortune, he made a failure. This feeling pursued him through the whole of his life. A little incident, of which he himself has furnished the particulars, strikingly illustrates the truth of this remark. One day, when hardly six years old, he was sent to borrow a little salt from one of the neighbors, an article which was then very scarce, and which cost at least twelve times as much then as now. It was a small quantity, tied up in a paper, which, when he was about half way home, tore, and out rushed the precious grains upon the ground. "As I write," says he, "nearly sixty years afterwards, the anguish which I felt at the sight seems almost to be revived. I had not then learned that the spilling of salt was portentous, but felt that it was a great present affliction." *

He was a man of extraordinary refinement. This feeling was deeply ingrafted in his constitution, and always

* Reminiscential Le.ters.

displayed itself, in a marked degree, in the presence of the female sex. Although his parents were uncultivated persons, and hardly ever mingled in refined society, they cherished as high and pure an idea of the duty of good breeding as any people on earth. The principle of politeness was deeply rooted in both, and they never failed to practice it in their family and in their intercourse with the world. *

An admiration for the female sex was one of the earliest sentiments developed in his moral nature. It swayed him through life, and continued to govern him to its close. "When that solemn event shall come," says he, "I hope to see female faces around my bed."—

> "And with a woman's hand to close
> My lids in death, and say—"Repose."

His mode of living was peculiar, and, in the opinion of the world and some of his friends, parsimonious and eccentric. Nothing, however, could have been more erroneous. The affection of his brain, which ultimately destroyed him, and to occasional attacks of which he was for many years subject, compelled him to live differently from other men. The slightest indulgence at dinner invariably brought on an attack of cerebral oppression, followed by an inability for useful mental and physical exertion; and it was a knowledge of this fact, the result of ample experience, that induced him never to take any thing at this meal, except a cup of tea and the smallest quantity of vegetables; frequently, indeed, nothing but a little pastry. At his breakfast and supper, however, he generally ate as heartily as any one. I allude to this subject, trivial as it may appear, and irrelevant as it may be to the true dignity of biography, because I wish to place my friend right before this community, in whose midst he lived and toiled for so many long winters. The explanation is due to his memory, to his children,

* Reminiscential Letters.

and to his friends. Boarding houses and hotels were disagreeable to him ; he could find no congeniality at a public table, and in the noise and confusion of public apartments. He preferred his own room at the University, with his cracker and cup of tea, to the most splendid table in the State. For many years he found a congenial place and a hearty welcome at the houses of his friends. It was not to save and hoard up money that he thus lived ; for no man ever spent money more liberally, no one ever had a greater contempt for it. His late associates in this University, and a few friends in this city, who alone knew him thoroughly and truly, can best appreciate the force and truth of my remarks.

To those who are engaged in scientific, literary, and educational pursuits, or in the practice of medicine, it will not be uninteresting to know that Dr. Drake was poor, and, until the last eight years of his life, pecuniarily embarrassed. It was not until after his connection with this University that he began to lay up any thing from his earnings. His medical journal only brought him into debt. The first volume of his great work has sold slowly, and had not yielded him one dollar at the time of his death. Since that period his son-in-law, Alexander H. McGuffey, Esqr., has received, as his literary executor, two hundred and fifty dollars as the balance to the author's credit up to that time. This sum is not more than one tenth of what he paid for the maps alone, contained in the work, and engraved at his own expense. Nothing, in fact, that Dr. Drake ever undertook was pecuniarily profitable. He was not a man of the money-making character. He lost money by every enterprise in which he ever engaged. His aims were always so lofty, and so far removed from self, that he never thought of money, except so far as it was necessary to their accomplishment.

But, although he has not, like Cæsar, left any landed estates, villas, orchards, or vineyards to his friends and the public, he has bequeathed them, what is far more precious and induring, a name without reproach, a bright example, and imperishable works.

Dr. Drake received, at various periods of his life, testimonials of the high appreciation of his professional character from different societies, both foreign and domestic. The names, with the date of their diplomas, of a few only of these are subjoined.

1. The Philadelphia Medical Society, March 1st, 1806. 2. The American Antiquarian Society, April 15th, 1818. 3. The American Philosophical Society at Philadelphia, April 17th, 1818. 4. The Wernerian Natural History Society of Edinburgh, Feb. 11th, 1826. 5. The Linnæn Society of Philadelphia. 6. The Medical Society of Massachusetts, July 10th, 1836. 7. The American Ethnological Society, Nov., 1842. 8. The Sweedish National Medical Society at Stockholm, Dec. 19th, 1848.

Only a fortnight before he died, he was elected an hon orary member of the Kentucky State Medical Society at its meeting in this city. Dr. Bartlett, Profesor of Materia Medica in the College of Physicians and Surgeons of New York, and Dr. Deaderick, of Athens, Tennessee, whose name is so honorably associated with the operation of excision of the lower jaw, were elected at the same time.

But the diploma which, of all others, he valued and appreciated most highly was the one already alluded to as having been given him by Dr. Goforth, at the close of his private pupilage. He was more proud of it, and felt more pleasure in recurring to it, than of any testimonial that could have been bestowed upon him by the Royal College of Surgeons of London, or the Royal Academy of Medicine of Paris. It was, as it were, his first professional love ; he had worked hard for it, and, no doubt, felt that he was worthy of it, conscious as he was of his deficiencies. This document, written on form paper, in a bold and beautiful hand, ran in this wise :

"Cincinnati, State of Ohio, August 1st, 1805.
I do certify
that
Mr. Daniel Drake
has pursued, under my direction,
for four years,
the study of Physic, Surgery, and Midwifery.
From his good abilities
and
Marked attention to the prosecution
of his Studies,
I am fully convinced that he is well qualified
to practise in these branches.
William Goforth, Jr.,
Surgeon-General
1st Divis. Ohio Militia."

How little he valued his other diplomas, is shown by the fact that he never referred to them in any of his publications. In his work on the diseases of the Interior Valley of North America his name appears without any titles, merely as Daniel Drake, M. D. I do not know that he was proud of this simplicity; probably he might have considered himself more honored by the breach than the observance.

Such, my associates, was the character of the physician, the teacher, and the medical philosopher, who, in the mellow twilight of evening, was struck from our ranks; and such, my fellow-citizens, was the character of the man, who, in the full vigor of his intellect, and in the hour of his usefulness, was struck from your midst. Truly, a great and good man has fallen. A great and noble intellect is extinguished. A bright and burning light has disappeared. But, although our friend is dead, yet does he live. He survives in his works and his example. His body has gone to the tomb, to moulder into dust, and return, atom by atom, to the earth from whence it was taken; his soul, purified and redeemed, has been restored to its God and its Creator; but all that was lofty, and refined and intellectual in his character will remain with us and with our posterity after us; gaining strength, and beauty, and freshness as it descends the stream of time. The good seed which he scattered upon the earth will continue to germinate and fructify as long as there shall be any soil for its reception, and the quickning of its living principle.

Shall it be said that such a man was born without an object; or that it does not matter whether he wrote such a work, or performed such a deed? The world, forgetful of its loss, and unconscious of his greatness, may move on in its unconcern as when he was in its midst; but that world would not have been complete without him. His presence was necessary to the age and generation in which he lived. Every human being, however humble or insignificant, is created for some given or specific purpose, although our feeble comprehension may not enable us to determine what that purpose is. Look at yonder star in the firmament. It is the smallest of its myriads of sisters, so small, indeed, as to be hardly visible to the unassisted eye, a mere speck, as it were, in the great celestial diadem, and yet, if removed from its orbit, or the place allotted to it, the whole machinery of the planetary system would instantly be thrown into a state of disorder and confusion. Will any one affirm that it was of no use, or that there was no design in its creation? The smallest pebble upon the sea shore, nay, the minutest grain of sand of which the imagination can conceive, is just as necessary to complete, and, for aught we know to the contrary, to uphold, the Universe as the mighty rock o Gibraltar, which rears its dark and frowning head aloft into the heavens from its broad ocean bed in the Atlantic Truth is eternal, and immutable; the works of the human mind never die; they form a part of the Universe, and are indispensable to the fulfilment of God's designs.

Dr. Drake lived in one of the most memorable epochs of the development of the human mind. The first half of the present century does not yield, in fame and usefulness, in great discoveries and improvements, to any period of time that has preceded it. His own profession has never advanced with a more steady and truly *progressive* pace. The lamp of medical science has never burned with a brighter and steadier flame. Discovery has followed discovery, and improvement improvement until the mind is absolutely bewildered by the scene before us.

Literature, science, and the arts, poetry, and philosophy, have never been more widely, or more successfully, cultivated. The blessings of knowledge, the arts of peace, and of civil and religious liberty have never been more extensively diffused among the nations of the earth. His own country has risen from an humble and feeble republic to the most exalted position that any government or people can occupy. The West, the more immediate theatre of his own fame and usefulness, has been transformed from a wilderness, the abode of the red man and the panther, into a smiling and luxuriant garden, covered with millions of inhabitants, and studded every where with cities, and towns, and villages, teeming with the arts, and luxuries, and refinements of civilized life. Its great waters, which, at the commencement of the present century, knew no vessels, save the Indian's canoe, and the adventurer's flat-boat, are now traversed by thousands of noble steamers, freighted with the rich and varied producc of our soil, and conveying the traveler in speed and safety to his destination.

The facilities of intercourse have been vastly increased. Fifty years ago a journey from Cincinnati to Philadelphia occupied the traveler between three and four weeks; now it can be performed in as many days. In 1800, there was not a single rail-road in the world; now there are thirteen thousand miles in the United States alone. Half a century ago it took nearly a month to convey intelligence from Cincinnati to New Orleans, or from the West to the Atlantic cities; now it requires hardly a few minutes. Then the lightning had been tamed; but it had not yet been taught to speak. Fifty years ago printing was performed entirely by hand ; now it is performed by steam, a single press being capable of throwing off twenty thousand newspapers an hour. When Dr. Drake entered the profession there was not a single medical school in the valley of the Mississippi ; now there are nearly twenty. Then there were few graduates of medicine ; now they are scattered over every portion of the country. He found his profession weak and obscure, and he left it in strength and beauty, having advanced it by his own labors, and adorned it by his own character.

But time admonishes me to bring this sketch to a close. Much more has already been said than the occasion requires, though much might be added that might be of interest and benefit to us all. In reviewing my labors I am conscious that they have fallen far short of what they ought to be, or of what they might have been in more able hands. From my intimate relations, personal and official, with the deceased, for a period of nearly twenty years, my colleagues, doubtless, concluded that the task of preparing a memorial of his life belonged more appropriately to me than to any one else. I can only say that I have endeavored to discharge the sacred trust, which their partiality has confided to me, to the best of my humble ability. It has been to me, throughout, a labor of love, not unmingled with the deepest sadness at the loss of him whose life and services we have this evening met to commemorate. I have endeavored to present a true picture of his character, and to speak of him as he was, and as he exhibited himself to us in his "daily walk and conversation." I have not indulged in panegyric, or fulsome eulogy. It has not been my object to weave a chaplet for his brow, an office of which he does not stand in need ; but to drop upon his grave, still fresh with the sod that covers it, a sprig of gnaphalium, as an emblem alike of our affection, and of his immortality upon earth.

